# Case Report: Analysis of Inflammatory Cytokines IL-6, CCL2/MCP1, CCL5/RANTES, CXCL9/MIG, and CXCL10/IP10 in a Cystic Fibrosis Patient Cohort During the First Wave of the COVID-19 Pandemic

**DOI:** 10.3389/fped.2021.645063

**Published:** 2021-07-06

**Authors:** Giulia Baresi, Mauro Giacomelli, Daniele Moratto, Marco Chiarini, Immacolata Claudia Conforti, Rita Padoan, Piercarlo Poli, Silviana Timpano, Francesca Caldarale, Raffaele Badolato

**Affiliations:** ^1^Pediatrics Clinic, “Angelo Nocivelli” Institute of Molecular Medicine, Azienda Socio Sanitaria Territoriale Spedali Civili of Brescia, Brescia University, Brescia, Italy; ^2^Flow Cytometry, Clinical Chemistry Laboratory, Azienda Socio Sanitaria Territoriale Spedali Civili of Brescia, Brescia, Italy; ^3^Cystic Fibrosis Regional Support Center, Azienda Socio Sanitaria Territoriale Spedali Civili of Brescia, Brescia, Italy

**Keywords:** SARS-CoV-2 infection, T cell activation, inflammatory activation, cystic fibrosis, cytokines

## Abstract

Since the beginning of the severe acute respiratory syndrome coronavirus 2 (SARS-CoV-2) pandemic, data registered in the European countries revealed increasing cases of infection in cystic fibrosis (CF) patients. In the course of this pandemic, we enrolled 17 CF patients for a study evaluating inflammatory markers. One of them developed COVID**-**19, giving us the possibility to analyze inflammatory markers in the acute phase as compared to levels detected before and after the infectious episode and to levels measured in the other CF patients enrolled to the study who did not experience COVID-19 and 23 patients referred to our center for SARS-CoV-2 infection.

## Introduction

Since the beginning of the severe acute respiratory syndrome coronavirus 2 (SARS-CoV-2) pandemic, data registered in the European countries revealed increasing cases of infection in cystic fibrosis (CF) patients with mild clinical manifestations of coronavirus disease 2019 (COVID-19) and usually favorable outcomes (1–4). This is surprising since due to their pulmonary impairment, CF patients are supposed to be considered subject to an increased risk of developing severe manifestations in case of SARS-CoV-2 infection. During the first wave of the SARS-CoV-2 pandemic (March to April 2020), we enrolled 17 CF subjects to evaluate the plasma levels of cytokines and chemokines. One of them developed COVID-19 during the study, giving us the opportunity to evaluate her immune and inflammatory response in the acute phase of SARS-CoV-2 infection.

## Case Report

We report on a 14-year-old girl affected by CF with pancreatic insufficiency followed at the Cystic Fibrosis Regional Support Center of the Pediatrics Clinic, University of Brescia, ASST Spedali Civili di Brescia (Brescia, Italy). She was diagnosed in the first days of her life, presenting with meconium ileus requiring surgery and CF newborn screening positive, being homozygous for the c.1521_1523delCTT (p.F508del) CF-causing mutation of the Cystic Fibrosis Transmembrane Conductance Regulator (*CFTR*) gene. During her follow-up, she developed chronic lung disease with bronchiectasis complicated by *Pseudomonas aeruginosa* intermittent respiratory infection. She was under treatment with pancreatic enzyme replacement therapy and multivitamin supplementation and received *CFTR* modulator therapy with lumacaftor/ivacaftor from July 2019. In March 2020, she was referred to our Emergency Department for persisting cough with purulent expectoration and dyspnea. At hospital admission, she presented good general conditions, apyretic (T 36.8°C) and tachypneic with oxygen desaturation (SpO2 93%). Physical examination revealed diffuse pulmonary crackles. Chest X-ray showed diffuse interlobular septal thickening with parenchymal infiltrates in the right hilus and the nasopharyngeal swab tested positive for SARS-CoV-2. The patient was hospitalized at the Pediatric COVID**-**19 Unit of the Pediatrics Clinic of Spedali Civili where she was treated with hydroxychloroquine 200 mg twice a day, according to our internal COVID**-**19 diagnostic and therapeutic protocol, broad-spectrum antibiotic therapy with cefotaxime, chest physiotherapy with positive expiratory pressure mask, and oxygen supplementation. Two days after the beginning of this therapy, the patient reported an improvement of pulmonary symptoms no longer requiring oxygen supplementation. During the hospitalization, she did not present fever or any other symptoms related to COVID**-**19. She was discharged after 10 days when two distinct nasopharyngeal swabs tested negative for SARS-CoV-2. After discharge, the patient continued the scheduled follow-up visits at the Cystic Fibrosis Regional Support Center. At the latest visit, she exhibited a FEV1 of 91% without presenting any complications related to the previous SARS-CoV-2 infection.

## Inflammatory Marker Analysis

At her hospital admission, the patient underwent laboratory tests, including blood counts, biochemical markers of inflammation (Table 1), plasmatic levels of cytokines [interferon-α (IFN-α), interferon-γ (IFN-γ), IL-1β, IL-4, IL-5, IL-6, IL-10, IL-12/p70, IL-17A, and tumor necrosis factor-α (TNF-α)] and chemokines (CXCL10/IP10, CXCL9/MIG, CCL5/RANTES, CCL2/MCP-1, and CXCL8/IL-8) (Table 2), and assessment of lymphocyte subsets, after informed consent registration obtained from both her parents (Study SARS-CoV-2-PED - NP4047 approved by Ethical Committee Spedali Civili, Brescia). Cytokine and chemokine plasma dosage has been performed with assay on plasma isolated from whole blood of each patient collected in EDTA, immediately frozen at −80°C in various aliquots and defrosted at the time of the analysis.

**Table 1:** Acute-phase reactants and serum biochemistry values of the index patient.

	**Patient**	**Normal values**
Red blood cells (×10^6^ cells/mmc)	4.42	4.5–10.0
White blood cells (×10^3^ cells/mmc)	6.42	4.5–10.8
Neutrophils (×10^3^ cells/mmc)	1.65	1.5–8.0
Lymphocytes (×10^3^ cells/mmc)	3.37	1.0–5.2
Hemoglobin (g/dl)	12.6	12.0–16.0
Platelets (×10^3^ cells/mmc)	390	100–400
C-reactive protein (mg/L)	0.4	<5
Serum amyloid A (mg/L)	2	<6.5
Ferritin (μg/L)	17	13–150
Troponin T(ng/L)	3	14
Lactate dehydrogenase (U/L)	218	120–300
D-dimer (ng/mL)	<200	<232
Fibrinogen (mg/dL)	260	100–410
Aspartate transaminase (U/L)	19	15–23
Alanine transaminase (U/L)	16	10–35

**Table 2:** Chemokine and cytokine levels of the CF patient were analyzed before SARS-CoV-2 infection, at the time of hospital admission (*t*_0_) and 30 days after discharge (*t*_30_).

	**Day 20/02/20**	***t*_**0**_ 31/03/20**	***t*_**30**_ 17/04/20**	**Healthy control (IQR range)**
	**(pg/ml)**	**(pg/ml)**	**(pg/ml)**	**(pg/ml)**
IFNγ	0.39	<0.1	<0.1	<0.1
IL-4	0.12	<0.1	0.2	<0.1
IL-5	0.64	0.64	<0.1	0.1–0.28
IL-10	2.13	<0.1	1.54	<0.1
IL-6	44.53	0.56	<0.1	0.1–0.51
CXCL8/IL-8	47.57	6.48	7.29	<0.1
CCL2/MCP-1	98.51	59.25	13.60	0.1–95.8
CCL5/RANTES	38.167	1,478	9,787	2,985–7,577
CXCL9/MIG	81.96	105.21	57.02	71.65–159.52
CXCL10/IP-10	260.57	104.03	40.07	61.06–130.05

*Healthy control ranges are shown as IQR range (pg/ml)*.

Plasma levels of CXCL10 (IP-10), CXCL8 (IL-8), CXCL9 (MIG), CCL5 (RANTES), and CCL2 (MCP-1) have been analyzed with flow cytometric bead array method, using the Human Chemokine Kit (Becton Dickinson, San Jose CA), following the manufacturer's instructions. IL-1β, IL-2, IL4, IL-5, IL-6, IL-10, TNF-α, IL-17A, IFN,-α, and IFN-γ have been dosed by Flex set custom cytometric bead array technique (Becton Dickinson). Afterward, data have been acquired on BD FACSCanto II flow cytometer and analyzed by FCAP v3 software.

**Figure 1:**
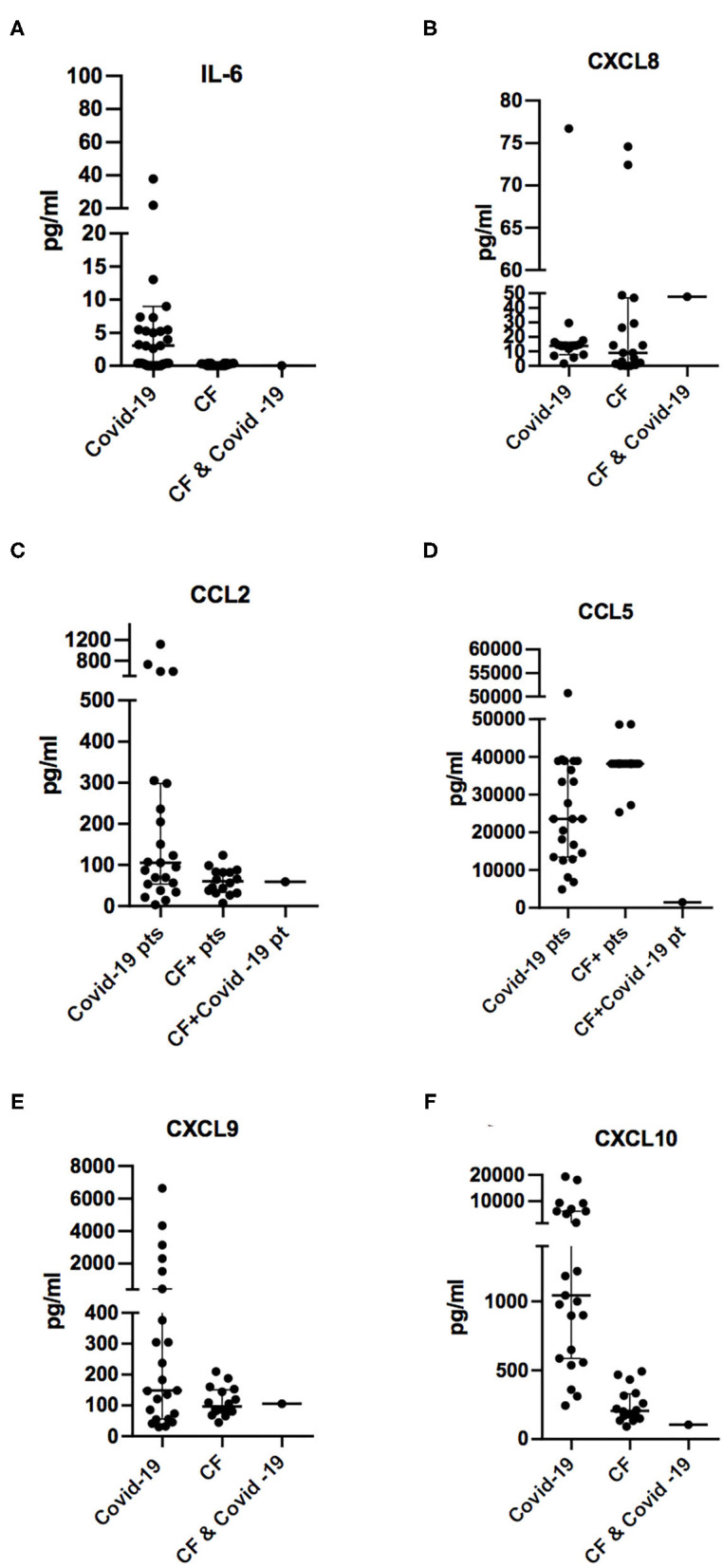
Comparison of plasmatic concentrations of chemokines and cytokines IL-6 **(A)**, CXCL8/IL-8 **(B)**, CCL2/MCP1 **(C)**, CCL5/RANTES **(D)**, CXCL9/MIG **(E)**, and CXCL10/IP-10 **(F)** from otherwise healthy subjects during SARS-CoV-2 infection (COVID-19), cystic fibrosis patients not infected with SARS-CoV-2 (CF), and the 14-year-old CF patient in the acute phase of viral infections (CF and COVID-19). Concentration levels are reported in the groups as median ± IQR (pg/ml).

Cytokine and chemokine values were compared with the levels measured in 23 children/young adults affected by COVID-19 (Study NP4000 approved by Ethical Committee Spedali Civili, Brescia) and with the levels measured in 16 subjects with CF (Study NP3087 approved by Ethical Committee Spedali Civili, Brescia evaluating cytokines and chemokines in CF patients) who were not affected by SARS-CoV-2 infection (Figure 1). These patients were previously enrolled in an ongoing study aimed to define inflammatory response in patients with CF.

CF patients presented a median age of 18.3 years without ongoing exacerbations at the time of blood samples, which were collected from May 2019 to February 2020. Ten out of 16 CF patients were female, and 14 presented pancreatic insufficiency, including three with type 1 diabetes mellitus. The median value of FEV 1 in the cohort was 83.65%. Six of them presented chronic inflammation due to *P. aeruginosa*. The other two patients showed infection by methicillin-resistant *Staphylococcus aureus* (MRSA) and one by *Achromobacter denitrificans*. All patients at the time of blood collection did not display active lung disease and specific infectious events and were not receiving immunomodulatory treatment or corticosteroids. Three CF subjects were under treatment with lumacaftor/ivacaftor at the time of blood draw.

Children with COVID-19 included 23 subjects (average age of 6.7 years). All patients were tested positive for SARS-CoV-2 at nasopharyngeal swab and presented mild or moderate course of COVID-19, without evidence of previous chronic respiratory diseases. All of them showed fever at the onset of symptoms, while only four patients manifested gastrointestinal symptoms. The other four patients reported influenza-like symptoms (rhinitis and cough). Chest X-ray showed interstitial thickening in 14 out of 23 cases, but only two children needed respiratory support: one with high-flow nasal cannula (HFNC) for a few days, and the other one with free-flow oxygen *via* facemask. Blood samples for plasma cytokine analysis were collected at the time of hospitalization prior to any treatment.

Because the index patient had been enrolled to study NP3087 before the occurrence of the SARS-CoV-2 pandemic, we had the opportunity to compare IL-6, CXCL8, CCL2, CCL5, CXC9, and CXCL10 plasmatic levels with those measured about 1 month before the viral infection. Because there are no standardized reference values for cytokines and chemokines, we analyzed for comparison the levels of cytokines and chemokines measured in 10 healthy control subjects (mean age: 45 years) and reported as median ± IQR (pg/ml) in Table 1.

Lymphocyte subpopulations, which were analyzed about 1 month before SARS-CoV-2 infection, at the time of acute infection, and 1 month after recovery, did not reveal significant changes of lymphocyte subsets, CD4^+^/CD8^+^ ratio, number of activated T cells, distribution of naïve, central memory, effector memory, and terminally differentiated CD4^+^ and CD8^+^ cells (data not shown). This is consistent with reports showing that children with SARS-CoV-2 infection do not display an increase of activation markers of T cell in the same extent observed in adults with COVID-19 (5).

## Comments

Since December 2019, a global effort is in progress in order to restrain the COVID-19 pandemic and identify the main pathophysiologic patterns of a disease that can drive severe complications. SARS-CoV-2 infection presents a broad spectrum of symptoms, ranging from influenza-like manifestations to severe progression toward pneumonia with acute respiratory distress syndrome, septic shock, cytokine storm syndrome, and coagulation dysfunction (6). CF (OMIM #219700) is a systemic disease caused by homozygous or compound heterozygous mutations in the CF transmembrane conductance regulator gene (OMIM ^*^602421) on chromosome 7q31 (7). Described as a triad of chronic obstructive pulmonary disease, exocrine pancreatic insufficiency, and elevated chloride concentration in sweat, the clinical phenotype can be heterogeneous, ranging from mild to severe forms of multi-organ disorders (8).

Reduced performance of CFTR channels leads to a pathogenic reduction in chloride and bicarbonate secretion and in the volume and pH of the airway surface liquid with subsequent abnormal mucous secretion and decreased mucociliary clearance (9). In addition, altered CFTR function affects the contribution of the epithelium to innate immunity through endogenous activation of nuclear factor κ light-chain enhancer of activated B cells (NF-κB) with subsequent anti-apoptotic and proinflammatory signaling by increasing expressions of IL-8 and granulocyte monocyte colony-stimulating factor (GM-CSF) (10, 11). Moreover, the surface expression of toll-like receptor-4 (TLR4) of epithelial cells in CF patients is reduced, resulting in decreased type I IFN regulation and proinflammatory signaling even in the absence of infectious trigger as well as exaggerated responses to microbial antigens (10–12).

The T-cell function appears to be also affected by defects in CFTR, and several studies have described reduced recruitment by CF epithelial cells of T cells in response to *S. aureus*, as well as altered T-cell responses to *Aspergillus fumigatus* in both human and murine models (10, 13, 14).

Recent studies of the host's immune response in SARS-CoV-2 suggest that early steps of the adaptive immune activity are connected with the formation of antigen-specific cytotoxic T cells and the synthesis of neutralizing antibodies (15–18). Although severe SARS-CoV-2 infection is characterized by extensive involvement of the adaptive immune system, the role of dysregulated innate inflammatory cytokine and chemokine responses is still to be confirmed as a contribution to acute COVID-19 severity (19). It has been reported that COVID-19 patients with severe outcome display higher plasma chemokines CXCL8/IL-8, CXCL9/MIG, and CXCL10/IP10, and cytokines IL-6 and IL-10 than patients with the mild disease (20), while children, who usually have a favorable outcome of SARS-CoV-2 infection, present lower levels of T-cell activation and pro-inflammatory cytokines (5). In particular, an increased level of proinflammatory cytokines, such as IL-2R, IL-6, IL-10, and TNF-α, has been associated with extensive lung damage and severe illness (21, 22).

To our knowledge, this is the first preliminary study that reports on the immunological characteristics of a patient affected by CF with laboratory-confirmed SARS-CoV-2 infection. The index patient presented with symptoms such as fever, cough, fatigue, and myalgia, which are reported to be the most common at the onset of the disease (23). Studies about laboratory markers of inflammation in COVID-19 disease support the evidence that leukocytosis (≥10 ×10^9^/L) and lymphopenia (<0.8 ×10^9^/L) were associated with an increase in blood concentrations of alanine aminotransferase (ALT) and aspartate aminotransferase (AST), lactate dehydrogenase (LDH), serum C-reactive protein (CRP), ferritin, and D-dimer, which are markedly higher in severe cases than in moderate ones (23, 24). This is consistent with our findings: the patients presented a mild course of infection, without any severe complication and any remarkable variations of acute-phase reactants and serum biochemistry values with subsequent rapid healing (Table 1). Case reports of CF patients with SARS-CoV-2 infection are increasingly described, and even though the CF population infected differs for age, sex, and variable pulmonary impairment with a wide range of complications and chronic respiratory infections, recent reports describe that the course of COVID-19 disease in CF appears to be milder than expected and less severe than casuistry of patients with other underlying lung diseases (9, 25, 26).

While most of the COVID-19 patients referred to our unit showed high levels of the proinflammatory cytokines and chemokines IL-6, CCL2, CCL5, CXC9, and CXCL10, low levels of these cytokines were detected in the blood of the 14-year-old CF patient during the SARS-CoV-2 infection.

In contrast, the neutrophil chemoattractant CXCL8/IL-8 was detected in the blood of CF patients at a slightly elevated level at the concentration range measured in CF patients without SARS-CoV-2 infection (Figure 1). This is consistent with the high levels of this chemokine, which are often detected in the blood of CF patients, especially in those with *P. aeruginosa* infection (27–29).

In the CF patient with COVID-19, we detected in the acute phase a lower concentration of CCL5/RANTES in comparison to chemokine levels measured in the same subject before the infection and in other CF patients not affected by SARS-CoV-2 (Figure 1 and Table 2). CCL5/RANTES is a chemokine produced by CD8^+^ T cells, epithelial cells, fibroblasts, and platelets, which plays a key role in the immune response to respiratory viral infections. Lower expression of CCL5/RANTES during SARS-CoV-2 infection in the CF patient might be related to the low levels of other proinflammatory cytokines or to a possible immunoregulatory role of CFTR channels on the host antiviral response.

Low blood concentrations of CXCL9/MIG and CXCL10/IP-10 might be a consequence of the low levels of T cell activation because the two chemokines are induced by IFN-γ, which is expressed by activated T lymphocytes, especially Type 1 helper (Th1) cells and natural killer (NK) cells during the immune response. Therefore, low levels of CXCL9 and CXCL10 in the CF patient affected by COVID-19 suggest that the antiviral response was not associated with Th1 polarization, which has been associated with an unfavorable outcome of COVID-19 in adult subjects (20).

Interestingly, in the index patient, the main inflammatory markers reported to be upregulated in SARS-CoV-2 infection (21, 30) appear to be even lower than the average measured in the CF patient without COVID**-**19 infection. This is surprising considering that in CF, the respiratory tracts are more susceptible to upregulated inflammation, particularly when triggered by new infectious agents (9, 31). In addition, because the CF patient was receiving treatment with lumacaftor/ivacaftor during COVID-19 episode, we speculate that treatment with CFTR modulators did not have a detrimental effect on the outcome of the disease and raises the question of the potential role of these drugs on regulating immune response or angiotensin-converting enzyme 2 (ACE2) expression. Although further studies are needed to answer these relevant questions, it is notable that levels of cytokines and chemokines in the other CF patients treated with lumacaftor/ivacaftor were in the same range as those in untreated CF patients.

A variety of variables have been considered as protective factors in CF patients affected with SARS-CoV-2 infection, such as the long-term use of antibiotics with anti-inflammatory or antiviral effects like azithromycin (32, 33). Moreover, it has been postulated that mutations in the CFTR gene may alter the protein expression of ACE2 through upregulation of vascular endothelial growth factor A (VEFGA) in such a way to mitigate the effects of infection on lung damage (9, 34). Furthermore, *in vitro* experiments on CF bronchial epithelial lines (CFBEs) showed no evidence that mutations in CFTR induce a hyperinflammatory response in CF epithelial cells (35), suggesting that other unknown regulatory mechanisms could be involved in the regulation of inflammation in CF cell lines. It has been presumed that epigenetic phenomena, such as DNA methylation or histone tail modification profiles, could modulate the gene expression and that could be cell-line specific, independent from F508 mutation (34).

## Conclusion

In conclusion, our report has revealed in a young CF patient with acute COVID-19 lower activation of the immune response as measured by levels of cytokines or chemokines such as IL-6, CCL2, CCL5, CXCL8, CXCL9, and CXCL10 than otherwise healthy pediatric patients, referred to our center for COVID-19. Interestingly, IL-6, CXCL8, CCL2, CCL5, and CXCL10 plasmatic concentrations were lower during the COVID-19 infectious episode as compared to levels measured 1 month earlier. These cytokines persisted low when re-evaluated after 1 month, suggesting that immune response against SARS-CoV-2 infection was not associated with increased inflammatory response.

Despite being a single case report, our experience suggests the possibility that SARS-CoV-2-related inflammatory disorders may express different clinical phenotypes depending on the pre-existing inflammatory and immune conditions.

## Data Availability Statement

The original contributions presented in the study are included in the article/supplementary material, further inquiries can be directed to the corresponding author/s.

## Ethics Statement

The studies involving human participants were reviewed and approved by NP3087, NP4000, NP4047. Written informed consent to participate in this study was provided by the participants' legal guardian/next of kin. Written informed consent was obtained from the individual(s), and minor(s)' legal guardian/next of kin, for the publication of any potentially identifiable images or data included in this article.

## Author Contributions

GB and RB: conceptualization and writing—original draft preparation. GB, MG, DM, MC, FC, IC, RP, PP, ST, and RB: data curation. GB, RP, PP, ST, and RB: writing—review and editing. RB: supervision, project administration, and funding acquisition. All authors contributed to the article and approved the submitted version.

## Conflict of Interest

The authors declare that the research was conducted in the absence of any commercial or financial relationships that could be construed as a potential conflict of interest.
